# Structure and Phase Composition of a W-Ta-Mo-Nb-V-Cr-Zr-Ti Alloy Obtained by Ball Milling and Spark Plasma Sintering

**DOI:** 10.3390/e22020143

**Published:** 2020-01-24

**Authors:** Ivan A. Ditenberg, Ivan V. Smirnov, Michail A. Korchagin, Konstantin V. Grinyaev, Vladlen V. Melnikov, Yuriy P. Pinzhin, Alexander I. Gavrilov, Maksim A. Esikov, Vyacheslav I. Mali, Dina V. Dudina

**Affiliations:** 1 Institute of Strength Physics and Materials Science of the Siberian Branch of the Russian Academy of Sciences, 634055 Tomsk, Russia; ditenberg_i@mail.ru (I.A.D.); smirnov_iv@bk.ru (I.V.S.); kvgrinyaev@inbox.ru (K.V.G.); pinzhin@phys.tsu.ru (Y.P.P.); 2 Department of Metal Physics, Faculty of Physics, National Research Tomsk State University, 634050 Tomsk, Russia; melnikov@phys.tsu.ru; 3 Institute of Solid State Chemistry and Mechanochemistry of the Siberian Branch of the Russian Academy of Sciences, 630128 Novosibirsk, Russia; korchag@solid.nsc.ru (M.A.K.); gavr_sand@mail.ru (A.I.G.); 4 Lavrentyev Institute of Hydrodynamics of the Siberian Branch of the Russian Academy of Sciences, 630090 Novosibirsk, Russia; esmax@yandex.ru (M.A.E.); vmali@mail.ru (V.I.M.)

**Keywords:** multicomponent alloys, powder mixture, refractory metals, ball milling, consolidation, spark plasma sintering, microstructure, microhardness

## Abstract

In this paper, the structural characteristics of a W-Ta-Mo-Nb-V-Cr-Zr-Ti non-equiatomic refractory metal alloy obtained by spark plasma sintering (SPS) of a high-energy ball-milled powder mixture are reported. High-energy ball milling resulted in the formation of particle agglomerates ranging from several tens to several hundreds of micrometers. These agglomerates were composed of micrometer and submicrometer particles. It was found that, during ball milling, a solid solution of A2 structure formed. The grains of the sintered material ranged from fractions of a micrometer to several micrometers. During SPS, the phase transformations in the alloy led to the formation of a Laves phase of C15 structure and ZrO and ZrO_2_ nanoparticles. The microhardness of the ball-milled alloy and sintered material was found to be 9.28 GPa ± 1.31 GPa and 8.95 GPa ± 0.42 GPa, respectively. The influence of the processing conditions on the structure, phase composition, and microhardness of the alloy is discussed.

## 1. Introduction

In recent years, multicomponent alloys and high-entropy alloys have received much attention, and currently represent a rapidly developing direction of materials science [1,2,3,4,5,6,7,8,9]. The interest in these alloys is due to unique combinations of mechanical properties that they can offer when properly designed. Research in this area is aimed at finding new promising compositions [1,2,3,6,7,8,9], characterizing the microstructure and mechanical properties [10,11,12,13,14,15,16], studying the mechanisms of plastic deformation [4,17], or determining the phase composition and phase stability of the alloys [18,19,20,21,22,23,24,25,26,27,28,29].

Multicomponent refractory alloys are interesting materials for nuclear power engineering as well as for aviation and space industries [2,7,10,13,16,24,25,28]. Investigations of these alloys are aimed at finding compositions that would ensure stable mechanical performance at elevated temperatures.

A serious metallurgical problem in the development of alloys containing both refractory and non-refractory metals is the significant differences in their melting points (for example, T_m_(W) = 3380 °C, T_m_(Ta) = 3014 °C, T_m_(V) = 1920 °C, and T_m_(Ti) = 1671 °C). For this reason, traditional metallurgy approaches do not work for these alloys.

One of the promising preparation methods of these alloys is ball milling [30] followed by spark plasma sintering (SPS) [31,32]. During ball milling, mixing of the non-interacting components occurs. In addition, low-temperature synthesis of compounds is facilitated in the ball-milled mixtures [30]. Furthermore, during high-energy ball milling, high local strains are achieved [33] such that the milled materials possess high concentrations of defects of the crystalline structure. Highly defect states are characterized by increased diffusion coefficients [34], which influence the process of the formation of new phases and sintering of the ball-milled powder mixtures. Sintering of the ball-milled mixtures by SPS is beneficial for conducting consolidation and synthesis at temperatures lower than those usually involved in conventional sintering. In contrast to cast alloys, the SPS processing excludes the formation of dendrite structures. Attempts to produce multicomponent refractory metal alloys have been described in [20,21,22,23,24,25,27,28].

In this work, the formation of W-Ta-Mo-Nb-V-Cr-Zr-Ti alloys by ball milling and SPS is presented. The goal of this study is to trace the evolution of the phase composition and microstructure of the alloy during milling and sintering and to determine the microhardness of the synthesized materials. A non-equiatomic chemical composition of the alloy was selected in an attempt to form a large number of phases in the material. The simultaneous nucleation of several phases can inhibit grain growth and make the dispersion hardening mechanism operate.

## 2. Materials and Methods

Metal powders of the following grades were used: W (PVCh, 99.48%, ≤5 μm), Ta (TaPA, 99.5%, ≤45 μm), Mo (MPCh, 99.5%, ≤5 μm), Nb (NB1, 99.98%, 10–63 μm), Zr (PTsrK1, 99.62%, ≤40 μm), Cr (PKh1S, 99.2%, 5–10 μm), V (VEL-1, 99.845%, ≤40 μm), and Ti (PTOM-2, 98.87%, ≤40 μm). The composition of the powder mixture is given in Table 1.

Ball milling of the powder mixture was conducted in a high-energy planetary ball mill AGO-2 with two water-cooled vials, each having a volume of 160 cm^3^. Stainless steel vials and balls were used. The diameter of the balls was 8 mm. The weight of the milling balls was 200 g, the weight of the sample was 12 g. The acceleration of the balls was 400 m s^−2^ (the rotational speed of the vials was 1500 rpm). Milling was conducted in an atmosphere of argon. The duration of milling under these conditions was 10 min. In order to avoid severe sticking of the powder to the vial walls and balls, after the powder was milled for 10 min, a process control agent was added into the vial (1 mL of ethanol) and milling was continued for another 30 s. After that, the vials were unloaded.

Sintering was conducted using a SPS Labox-1575 furnace (Sinter Land Inc., Nagaoka, Japan) at a temperature of 1250 °C and a uniaxial pressure of 40 MPa in forevacuum (residual pressure 10 Pa). The holding time at the maximum temperature was 5 min. The heating rate up to the maximum temperature was 100 °C min^−1^. The average cooling rate, after the current was switched off, was 80 °C min^−1^.

The morphology and elemental composition of the ball-milled powders as well as the microstructure of the sintered alloy were examined by scanning electron microscopy (SEM) using a FEI Quanta 200 3D scanning electron microscope operating at 30 kV and equipped with a focused ion beam (FIB) instrument. Energy-dispersive spectroscopy (EDX) was used to study the distribution of the elements in the alloy.

The phase composition of the synthesized alloys was determined by X-ray diffraction (XRD) on a Shimadzu XRD 6000 diffractometer using Cu Kα radiation. The phase identification was performed using PDF-4 database. POWDER CELL 2.4 software was used for the full-profile analysis of the XRD patterns.

Transmission electron microscopy (TEM) was used to investigate the fine structure of the alloys. The analyses were conducted using a Philips CM30 TWIN microscope operating at 300 kV and a Philips CM-12 microscope operating at 120 kV. For the local elemental analyses, a Jeol 2100 (200 kV) microscope was used. Samples for TEM observations were obtained using FIB (FEI Quanta 200 3D) and two-sided ion milling (by argon ions) using a Gatan device operating at a voltage of 5 kV.

The Vickers microhardness of the alloys was measured using a Neophot 21 device under a load of 0.5 N using a holding time of 15 s. The microhardness of the sintered alloys was measured on the polished bulk samples, while that of the ball-milled alloy was measured on the polished cross-sections of the powder agglomerates mounted in epoxy resin.

## 3. Results

**Figure 1:**
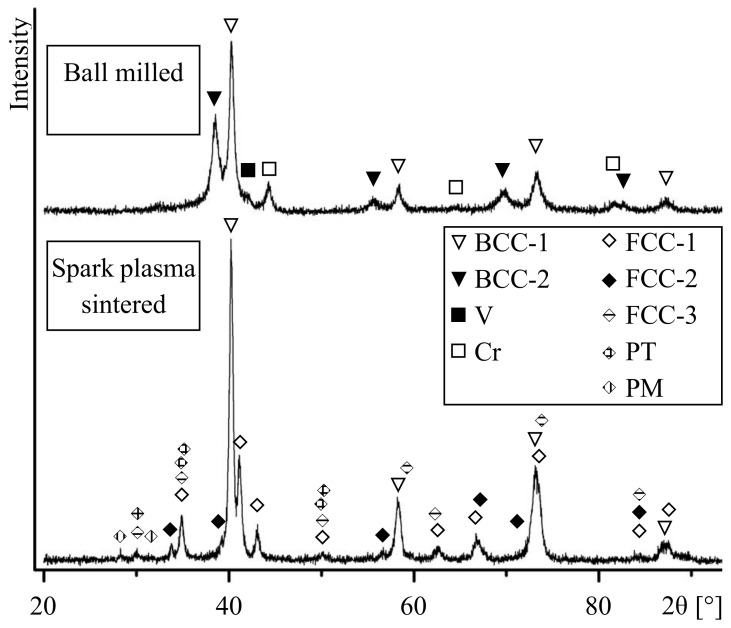
X-ray diffraction patterns of the W-Ta-Mo-Nb-V-Cr-Zr-Ti alloy after ball milling and spark plasma sintering (SPS) (BCC, body-centered cubic; FCC, face-centered cubic; PT, tetragonal phase; and PM, monoclinic phase).

Figure 1 shows the XRD patterns of the ball-milled and sintered alloys. The patterns show broadened reflections of the phases, which is due to the crystallite size refinement and lattice strain; the concentration gradients in solid solutions can also contribute to line broadening [35]. Some reflections of the metallic components are no longer detected in the pattern. In particular, reflections of metallic zirconium are not visible in the pattern. The presence of metallic chromium is confirmed by its (110), (200), and (211) reflections. The ball-milled alloy contains two body-centered cubic (BCC) phases of A2 structure designated as BCC-1 and BCC-2. Reflections of BCC-1 phase with (110), (200), (211), and (220) indices are detected corresponding to interplanar spacings of 2.24 Å, 1.58 Å, 1.29 Å, and 1.12 Å. Reflections of BCC-2 phase are from planes with the same indices and correspond to 2.33 Å, 1.65 Å, 1.35 Å, and 1.17 Å interplanar spacings.

According to the binary phase diagrams [36], W, Ta, Mo, Nb, V and Ti can form continuous series of solid solutions with BCC lattice. The lattice parameter of BCC-1 is close to those of W (a = 3.165 Å), Mo (a = 3.147 Å), WMo (a = 3.155 Å), TaV (a = 3.175 Å), NbV (a = 3.18 Å) and Mo_0.6_Ti_0.4_ (a = 3.199 Å). Therefore, BCC-1 is a disordered W-Ta-Mo-Nb-V-Ti solid solution formed as a result of mixing during ball milling. The positions of the broadened reflections of BCC-2 are close to those of Ta (a = 3.303 Å), Nb (a = 3.295 Å), Ti_β_ (a = 3.30 Å), NbTi (a = 3.286 Å), and Nb_2_Mo_2_Zr (a = 3.27 Å). It is known that Mo, Nb, and Ta favor the Ti_α_ → Ti_β_ transition; upon quenching, Ti_β_ solid solution can be stabilized. Therefore, BCC-2 can be a substitutional solid solution of Ta-Mo-Nb-Ti-Zr composition. Along with reflections of BCC-1 and BCC-2 phases, those of metallic chromium are observed. The absence of reflections of metallic zirconium can be explained by the formation of BCC-2 and partial dissolution of zirconium in BCC-1. Furthermore, a Laves phase of C15 structure, Mo_2_Zr (a = 7.585 Å) or W_2_Zr (a = 7.621 Å), can form in small concentrations. Due to overlapping of reflections, it is not possible to distinguish the lines of these phases in the pattern.

In the XRD pattern of the sintered alloy, reflections of BCC-2, Cr, and V are no longer observed. At the same time, the intensities of reflections of BCC-1 increase (Figure 1). This indicates a higher content of BCC-1 in the sintered alloy as compared with the ball-milled mixture. In addition, reflections of a monoclinic phase (PM), a tetragonal phase (PT), and three face-centered cubic phases (FCC-1, FCC-2, and FCC-3) are detected.

The diffraction maxima of FCC-1 (220), (311), (222), (422), (511), and (440) correspond to interplanar spacings of 2.57 Å, 2.19 Å, 2,10 Å, 1.48 Å, 1.40 Å, and 1.29 Å, respectively. This indicates that FCC-1 has C15 structure, which is a cubic modification of a Laves phase and has a lattice parameter of a = 7.27 Å, which is close to that of Cr_1.2_V_0.8_Zr (a = 7.28 Å) and Cr_1.8_Mo_0.2_Zr (a = 7.244 Å).

Lattice spacings corresponding to (111), (200), and (220) reflections of FCC-2 are 2.65 Å, 2.30 Å, and 1.62 Å, respectively. The (111) and (220) diffraction maxima of FCC-3 correspond to 2.97 Å and 1.82 Å. The FCC-2 and FCC-3 phases possess A1 structure with lattice parameters of a = 4.59 Å and a ≈ 5.15 Å, respectively. These phases can, therefore, be identified as zirconium monoxide ZrO (a = 4.602 Å) and zirconium dioxide ZrO_2_ (a = 5.135 Å).

The diffraction maxima of PT phase (110), (002), (110), (112), and (200) correspond to interplanar spacings of 2.97 Å, 2.60 Å, 2.57 Å, 1.83 Å, and 1.81 Å, respectively. This lattice can be described as P42/nmc with parameters a ≈ 3.63 Å and c ≈ 5.20 Å, which are close to tetragonal ZrO_1.97÷2_ (a ≈ 3.63 to 3.64 Å and c ≈ 5.20 to 5.23 Å). Reflections (-111) and (111) of a monoclinic phase correspond to lattice spacings of 3.16 Å and 2.84 Å. Therefore, the monoclinic lattice can be described as P21/c with the following parameters: a ≈ 5.15 Å, b ≈ 5.21 Å, c ≈ 5.31 Å, and β ≈ 99.23°. The parameters of PM are close to monoclinic ZrO_2÷2.12_ (a ≈ 5.12–5.15 Å, b ≈ 5.20–5.21 Å, c ≈ 5.28–5.32 Å, and β ≈ 99.10–99.24°).

**Figure 2:**
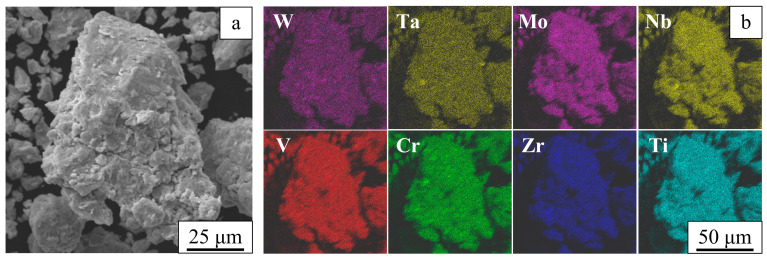
An image of a powder agglomerate recorded in the topographic contrast (**a**) and chemical element distribution maps (**b**). Ball-milled W-Ta-Mo-Nb-V-Cr-Zr-Ti.

The ball-milled alloy consists of powder agglomerates ranging from several tens to several hundreds of micrometers in size (Figure 2a). These agglomerates consist of submicrometer particles. The elemental mapping shown in Figure 2b confirms uniform mixing of the alloy components.

**Figure 3:**
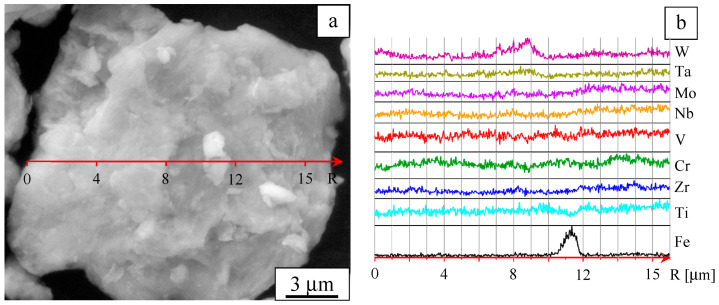
Microstructure of a powder agglomerate (**a**) and element distribution profiles (**b**). Ball-milled W-Ta-Mo-Nb-V-Cr-Zr-Ti.

The microstructure of the alloy particle (agglomerate) is presented in Figure 3a. The concentration profiles of the elements are shown in Figure 3b. It can be seen that some areas feature increased concentrations of the components. In addition, it was found that the ball-milled alloy contains iron at a concentration of about 2 at%; iron is present in the form of separate particles. Iron contamination is caused by wear of the milling media and vials.

The choice of the milling duration for the preparation of the alloy was dictated by two factors: achieving uniform mixing and keeping the iron contamination at a low level. Milling of the powder mixture for durations longer than 10 min led to a rapid increase in the iron contamination of the powder. In mixtures milled for shorter durations, uniform mixing was not yet achieved.

**Figure 4:**
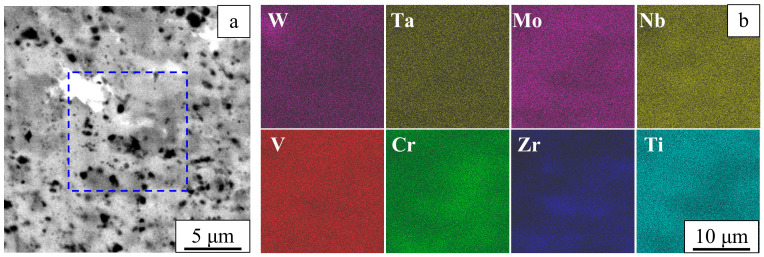
Composite microstructure in Z-contrast (**a**) and element distribution maps (**b**). Sintered W-Ta-Mo-Nb-V-Cr-Zr-Ti alloy.

A Z-contrast image of the sintered alloy (Figure 4a) shows four regions of different contrast: white, gray, dark-gray, and black. These regions differ in the chemical compositions. In the sintered alloy, variations of the chemical composition are detected on the micrometer and submicrometer scale (Figure 4b).

**Figure 5:**
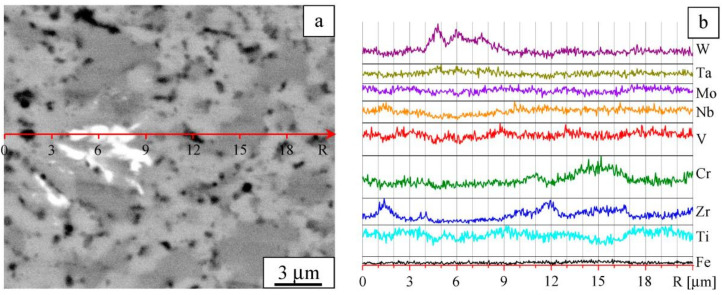
Details of the microstructure: Image taken in Z-contrast (**a**) and element distribution profiles (**b**). Sintered W-Ta-Mo-Nb-V-Cr-Zr-Ti alloy.

**Figure 6:**
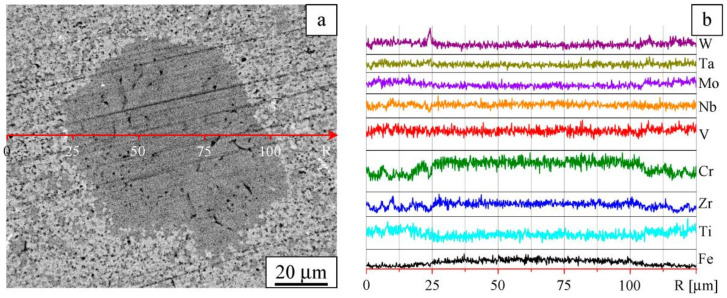
Microstructure in Z-contrast (**a**) and element distribution profiles (**b**). Sintered W-Ta-Mo-Nb-V-Cr-Zr-Ti alloy, area showing compositional non-uniformity.

Microstructures of different areas of the sintered alloy sample are shown in Figure 5a and Figure 6a. The latter demonstrates an area of non-uniform chemical composition. The profiles of the elements are shown in Figure 5b and Figure 6b, respectively.

The analysis of the XRD data together with results of SEM/EDX allows us to assign BCC-1 phase to gray regions, as shown in Figure 5a. According to Vegard’s law, a_mix_ = ∑C_i_ a_i_ [37]. Therefore, the lattice parameter of the material corresponding to these regions can be estimated as follows: a_BCC-1_ = ∑C_i_a_i_ ≈ 3.17 Å (i = W, Ta, Mo, Nb, V, Cr, Ti). The calculated lattice parameter agrees well with the lattice parameter determined from the XRD analysis. The concentration of BCC-1 is about 65%, and, therefore, BCC-1 can be regarded as a matrix of the alloy.

In the white regions, the concentration of W is twice its concentration in BCC-1 (Figure 5a,b). The sizes of these regions range from fractions of a micrometer to several micrometers (Figure 4a and Figure 5a). The concentration of the W-rich regions is not high and does not exceed 0.3 vol%. Due to peak overlapping in the XRD patterns, it does not appear possible to determine the specific features of the structural state of these regions.

The dark-gray regions are enriched with Zr and Cr, as can be concluded from Figure 4, Figure 5 and Figure 6. These regions can be assigned to FCC-1, which contains Cr, Zr, V, and Ti as the major components and Nb, Ta, W, and Mo as the minor components. Occasionally, FCC-1 is found in the form of equiaxed particles up to 100 μm in size (Figure 6a). Iron introduced into the alloy during milling is present in both small and large particles of FCC-1. On the basis of the XRD data and SEM observations, the volume fraction of FCC-1 was estimated to be 30%.

Black spots in the SEM images are smaller than 1 μm and correspond to equiaxed particles. These particles show the highest concentration of Zr among the constituents of the alloy and are distributed mainly along the boundaries between BCC-1 and FCC-1 phases (Figure 5a and Figure 6a). Results of XRD and EDX indicate that these black spots are ZrO- and ZrO_2_-based particles and correspond to different phases (FCC-2, FCC-3, PT, and PM). The total volume fraction of these phases is about 3%. The major contribution comes from FCC-2, which is represented by relatively large particles (up to 1 μm) of ZrO.

**Figure 7:**
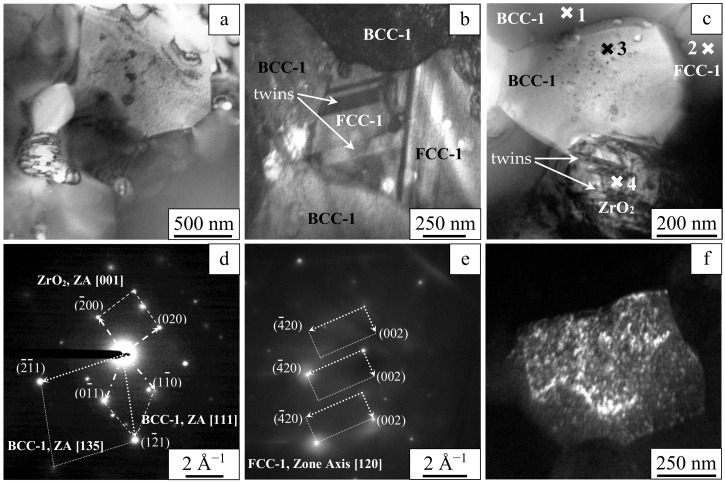
Transmission electron microscopy of the W-Ta-Mo-Nb-V-Cr-Zr-Ti alloy processed by SPS. Bright-field images of the grain and heterophase structure (**a**–**c**), microdiffraction patterns (**d**,**e**), and dark-field image of finely dispersed particles of inclusion (**f**).

TEM investigations have shown that the sintered alloy consists of submicrometer and micrometer grains (Figure 7). Equiaxed particles 200 to 400 nm in size are also found (Figure 7a,c). In addition, faceted particles several tens of nanometers in size can be seen at the grain boundaries and within the grains (Figure 7a–c).

Dark-field imaging [38] allowed detecting nanotwins in the Laves phase. The width of these nanotwins ranges from several tens of nanometers to several hundreds of nanometers. The grains of BCC-1 showed speckled diffraction contrast (Figure 7b,c), which is normally observed in heterophase states [38]. In large particles, nanotwins with widths not exceeding 40 nm were found (Figure 7c).

Table 2 shows the results of the local elemental analysis of areas marked 1, 2, 3, and 4 in Figure 7c. It can be seen that the elemental composition of BCC-1 and FCC-1 differ significantly by the concentration of Zr and Cr. Large particles (Area 4, Figure 7c) are rich in Zr. In grains of BCC-1, some regions are Zr-enriched (for example, Area 3). The cross-sectional area of the electron beam was ~80 nm^2^.

The analysis of the electron diffraction patterns (see example in Figure 7d,e) shows that materials of the grains belonging to the same phase can have slightly different lattice parameters due to local differences in the chemical composition. In particular, the lattice parameter of BBC-1 varies from 3.05 to 3.20 Å, while the lattice parameter of FCC-1 is between 7.24 and 7.28 Å. Particles 200 to 400 nm in size appear to represent monoclinic zirconia with lattice parameters a ≈ 5.12–5.15 Å, b ≈ 5.20–5.21 Å, c ≈ 5.28–5.32 Å, and β ≈ 99.10–99.24°. Zirconia particles having sizes of several tens of nanometers represent the tetragonal phase with lattice parameters a ≈ 3.63–3.64 Å and c ≈ 5.20–5.23 Å. Dark-field imaging detects particles less than 10 nm in size (Figure 7f). These particles are distributed in the grains of BCC-1 and correspond to cubic zirconia (FCC, a ≈ 5.13–5.15 Å). 

Table 3 shows the microhardness values of the synthesized alloy. An average microhardness of the sintered alloy is slightly lower than that of the ball-milled alloy. The microhardness of the sintered alloy shows a smaller standard deviation of the value than that of the ball-milled alloy. The latter indicates a higher uniformity of the microstructure of the sintered alloy in comparison with the ball-milled alloy.

## 4. Discussion

A common feature of the phase evolution process of multicomponent alloys obtained by solid-state processing is the formation of a “matrix” phase, which is a disordered substitutional solid solution of BCC structure, at the mixing and alloying stage (during ball milling) [20,21,23,24,25,27,28]. In this work, the use of a high-energy ball mill dramatically reduced the milling time as compared with other studies. Indeed, the ball milling time can be several hours [23,25,27,28] or several tens of hours [20,21,24,25,27,28] depending on the type of milling equipment used (Table 4).

In addition to mixing, treatment in high-energy ball mills results in severe deformation of the materials and the formation of highly defect nanostructured states [39,40]. The formation of such states results in the reduction of the sintering temperature of the powders owing to increased diffusion coefficients of the components of the alloy [34]. It should be noted that, in many investigations, SPS temperatures were higher than in the present work (1400–1700 °C, Table 4).

A significant outcome of applying a low sintering temperature is the synthesis of a fine-grained W-Ta-Mo-Nb-V-Cr-Zr-Ti alloy. The microhardness of the alloy synthesized in the present work is comparable to that of alloys synthesized by other researchers (Table 4). The strength of these alloys depends on a number of factors, which include grain refining [21,23,24,27], the fraction of Laves phase [21,23,25,28], the presence of reinforcing particles of other phases [20,21,24,25,27,28], and solid-solution strengthening [20,27,28].

In the alloy studied in this work, the non-equiatomic composition favors the formation of a high fraction of Laves phase, which is different from the equiatomic systems [21,23,25,28]. In particular, Laves phases are favored by high concentrations of Zr, Cr, V, and Ti [23,26,28].

High chemical activity of refractory metals [41] often causes the formation of composite (heterophase) materials. In powder systems subjected to ball milling, the presence of interstitial atoms of contaminants in the lattice (e.g., oxygen originating from films covering the surface of the powder particles) facilitates the nucleation of particles of new phases. The detection of very fine particles of ZrO_2_ within the grains of BCC-1 phase indicates that Orowan mechanism can be operative in this material. It should be noted that Orowan strengthening is one of the efficient strengthening mechanisms of refractory metals [42,43,44]. Cubic and tetragonal modifications of ZrO_2_ are stronger [45] and possess high elastic moduli [46] as compared with the monoclinic ZrO_2_ phase. These factors can be beneficial for dispersion strengthening of metallic matrices.

In this connection, a question of stability of fine particles distributed in a matrix is very relevant. It is known that cubic zirconia exists at temperatures exceeding 1900 °C [47]. In very fine particles, the boundaries of phase stability can shift due to the contribution of an increased surface energy of the system [48]. Stabilization of cubic zirconia at low temperatures can be caused by the presence of alloying additives (for example, carbon as an interstitial impurity and Ti or Nb as a substitutional impurity [47]).

In [49], stabilization of cubic zirconia in the form of nano-sized particles was observed in V-based alloys dispersion-strengthened by internal oxidation. The presence of dispersed particles causes increased short-term high-temperature strength and improved high-temperature phase stability of the alloy. Furthermore, in [50], it was shown that nano-sized ZrO_2_ particles stabilize grain boundaries enhancing the Hall–Petch effect and help retain the fine-grained state of the material at elevated temperatures. Therefore, in alloys containing dispersed particles, mechanical strength should be considered as a sum of different contributions. Along with grain refinement and the presence of Laves phases, it is necessary to consider strengthening by the formation of subgrains and dispersion strengthening caused by the presence of particulate inclusions in a BCC matrix.

## 5. Conclusions

High-energy ball milling of a W-Ta-Mo-Nb-V-Cr-Zr-Ti alloy with a non-equiatomic composition results in the formation of a BCC matrix, which is a disordered substitutional solid solution. The alloy obtained by SPS of the ball-milled powders exhibits a composite structure and consists of submicrometer and micrometer grains. The non-aquiatomic composition of the alloy leads to the formation of a high volume fraction of Laves phase (30%) of FCC structure along with the BCC matrix. The submicrometer grains of the Laves phase show nanotwins with widths from several tens to several hundreds of micrometers. The sintered alloy contains zirconium monoxide and dioxide particles (up to 3 vol%). Micrometer ZrO particles and submicrometer ZrO_2_ in monoclinic and tetragonal modifications are distributed along the grain boundaries of the BCC matrix and the FCC Laves phase. Within the grains of the BCC matrix, nano-sized particles of cubic zirconia are located. This work shows that, in refractory metal-based alloys, multiple strengthening mechanisms should be taken into account. Along with grain refinement and the presence of Laves phases in high volume concentrations, strengthening can be due to the formation of subgrains and the presence of inclusions in a BCC matrix.

## Figures and Tables

**Table 1:** Elemental composition of the powder mixture.

Composition	W	Ta	Mo	Nb	Zr	Cr	V	Ti
at%	5.3	5.4	10.1	10.5	10.7	18.7	19.1	20.3

**Table 2:** Local elemental composition (at%) of areas marked 1, 2, 3, and 4 in Figure 7c (EDX analysis).

Area	W	Ta	Mo	Nb	Zr	Cr	V	Ti
1 (BCC-1)	9	8	13	14	3	13	17	23
2 (FCC-1)	6	8	5	9	16	29	16	11
3 (FCC-2, FCC-3)	2	3	1	3	78	2	1	10
4 (FCC-3)	3	5	0	4	81	3	2	2

**Table 3:** Microhardness of the W-Ta-Mo-Nb-V-Cr-Zr-Ti alloy processed by ball milling and SPS.

Processing	Hμ (GPa)
Ball-milled	9.28 ± 1.31
Spark Plasma Sintered	8.95 ± 0.42

**Table 4:** Composition, processing conditions, and microhardness of refractory metals-based multicomponent alloys.

System	Processing	Hμ (GPa)	Reference
W-Ta-Mo-Nb-V-Cr-Zr-Ti	10.5 min milling and SPS (1250 °C)	8.95	This work
W-Ta-Mo-Nb-V-Cr	40 h milling and SPS (1400–1700 °C)	10.52	[20]
WMoNbCrTi	5–40 h milling and SPS (1400 °C)	10.40	[23]
W-Mo-Nb-Cr-Ti	7–10 h milling and SPS (1100–1300 °C)	8.9	[25]
W0.3(TaTiCrV)0.7	3 h milling and SPS (1600 °C)	8.4 (790 Hv)	[28]
W-Ta-Mo-Nb	24 h milling and SPS (1600 °C)	7.78	[24]
NbMoTaWVTi	40 h milling and SPS (1400 °C)	–	[21]
WNbMoTaV	6 h milling and SPS (1500–1700 °C)	–	[27]
